# Effect of Phosphate Starvation on Gene Expression in *Komagataella phaffii* Cells

**DOI:** 10.3390/microorganisms13010039

**Published:** 2024-12-28

**Authors:** Valeria V. Ishtuganova, Anton V. Sidorin, Anastasiya S. Makeeva, Marina V. Padkina, Andrey M. Rumyantsev

**Affiliations:** Department of Genetics and Biotechnology, Saint Petersburg State University, 199034 St. Petersburg, Russia; valeriishtuganova@gmail.com (V.V.I.);

**Keywords:** *Komagataella phaffii*, phosphorus, transcriptomic analysis, differential gene expression

## Abstract

Phosphorus is a key nutrient for all organisms. The study of phosphate metabolism and its regulation is important for understanding the evolutionary processes of regulatory systems in eukaryotic cells. The methylotrophic yeast *Komagataella phaffii* is an efficient producer organism, and it is actively used in biotechnological production. The high practical importance of *K. phaffii* has stimulated active research to find new tools to work with this yeast and optimize its cultivation conditions. In this work, we observed the effect of phosphate starvation on gene expression in *K. phaffii* at the transcriptome level. Phosphate starvation had a significant effect on general cell metabolism. *K. phaffii* cells demonstrated a response to this macronutrient deficiency through an altered gene expression of carbon and amino acid metabolism. We observed the activation of phosphate and polyphosphate metabolism gene expression. In this case, there was a suppression of ribosome biogenesis genes and genes involved in fatty acid beta-oxidation and translation processes.

## 1. Introduction

In the past, studying organisms at the molecular level was a difficult and time-consuming task. Previously, research has focused on individual genes and required many financial and time-related resources. Moreover, studies were performed on classical model objects of genetics and molecular biology. The development of molecular biology has generated new opportunities for scientists. Modern methods make it possible to study individual groups of genes, their protein products, and entire biochemical pathways in detail. The range of model objects available has rapidly expanded and been supplemented with non-traditional organisms. The active study of new organisms has been stimulated by their high practical significance. One example is the biotechnologically important yeast *Komagataella phaffii.*

The methylotrophic yeast *K. phaffii* is widely used as an efficient producer of various recombinant proteins. It is able to achieve high culture densities on simple media and demonstrate high levels of target protein synthesis. Expression systems based on the yeast *K. phaffii* have proven to be efficient and convenient platforms for the synthesis of various substances. More than 5000 pharmaceutical and recombinant proteins have been produced using *K. phaffii* yeast [1]. It is also actively used for the production of antibody fragments, vaccines, food components, and chemical compounds [2,3,4].

The main feature of the yeast *K. phaffii* is the presence of extremely strong and highly regulated promoters of methanol metabolism genes (*PAOX1*, *PDHAS*) [5]. Due to the high toxicity and flammable nature of methanol, there are restrictions to using these promoters for the production of recombinant proteins. Therefore, the research is focused on finding new tools for working with these microorganisms to increase their efficiency as recombinant protein producers.

The high practical importance of the yeast *K. phaffii* has stimulated active study on the peculiarities associated with its biochemical processes. The yeast is now actively considered an excellent model organism for various types of fundamental research. The mechanisms of the cell cycle, ribosome and peroxisome biogenesis, and mitochondrial function have been studied using these microorganisms [6,7]. The yeast cells finely regulate biochemical processes in macro- and micronutrient deficiencies. The gene regulation and metabolic pathways of *K. phaffii* in response to carbon and nitrogen, vitamin, and amino acid starvation are being actively researched [8,9]. However, the effects of phosphate deficiency on *K. phaffii* metabolism remain poorly understood.

Phosphorus is an important chemical element. It plays a central role in the vital activity of all organisms. Phosphorus is part of nucleotides, ATP molecules, and phospholipids, and it plays an important role in signal transduction and participates in the coordination of cellular metabolism. It is also found in cells as salts and phosphoric acid esters. Some biomolecules containing phosphate groups serve as energy sources, helping in the maintenance and regulation of many biochemical processes in cells [10,11].

Phosphate metabolism and mechanisms of its regulation have been studied extensively in a well-known model object: the yeast *Saccharomyces cerevisiae* [12]. Phosphate metabolism and mechanisms of its regulation are complicated and multicomponent processes. The key components of phosphate metabolism are controlled by regulatory elements of the PHO pathway. This signaling pathway allows *S. cerevisiae* yeast cells to coordinate cellular biochemical processes and quickly adapt to changes in the phosphate concentration in the medium [13].

The main regulators of the PHO pathway are the transcription factor Pho4p and the protein kinase complex Pho80-85p. Pho4p can be transported into the nucleus. At high phosphate concentrations, the Pho80-85p complex phosphorylates the transcription factor. In this case, Pho4p is rapidly exported from the nucleus and does not activate the transcription of phosphate metabolism genes [14]. Under low-phosphate conditions in a cell, the complex of the cyclin Pho80p and the cyclin-dependent kinase Pho85p is unable to efficiently phosphorylate the transcription factor [15]. Pho4p binds to the promoter region of PHO genes and initiates their transcription. Consequently, the expression of phosphatase genes, inorganic phosphate transporters, and the genes involved in polyphosphate metabolism are activated at low phosphate concentrations.

As previously mentioned, the phosphate metabolism in the yeast *K. phaffii* has not been studied extensively. Therefore, it is particularly interesting to study these microorganisms in comparison with other researched species. The development of omics technologies has made it possible to obtain large amounts of data on various processes in different yeasts [16]. A comparison of metabolic pathways and the mechanisms behind their regulation in “non-conventional” organisms is important for understanding evolutionary processes, and also contributes to better understanding and identification of common and different regulatory mechanisms. We hypothesize that *K. phaffii* yeast cells will show a similar response to phosphate starvation to *S. cerevisiae* yeast cells. However, due to the peculiarities of their metabolic processes, we also expect to find specific response characteristics of *K. phaffii* yeast cells.

In the presented work, we study the response of *K. phaffii* yeast cells to phosphate deficiency in the medium using transcriptome analysis.

## 2. Materials and Methods

### 2.1. Media and Cultivation Conditions

*K. phaffii* yeast strains (Thermo Fisher Scientific, Waltham, MA, USA) were routinely grown on YPDs media. Cells were cultured in MFI media for transcriptome analysis. One liter of YPDs contained 2% D-glucose, 2% peptone, 1% yeast extract, and 18.2% sorbitol (2.4% agar was also added to make solid medium). One liter of MFI medium contained sodium-citrate buffer (pH = 5.5; 0.1 M), salt solution (MgSO_4_·7H_2_O—12.5 g, (NH_4_)_2_SO_4_—7.2 g, CaCl_2_ (anhydrous)—1.44 g), 1 g/L KH_2_PO_4_, vitamins, and trace elements—400 μL/mL of 2500× solutions (Table S1 in the Supplementary Materials); with carbon source as 1% glycerol.

MFI media (600 mL) with glycerol were inoculated with the *K. phaffii* X-33 strain. After 40 h of cultivation, two equal 300 mL samples were taken. Cells were collected using centrifugation (10 min at 5000 rpm). One sample was resuspended in 300 mL of the media without phosphate source, and the other was resuspended in 300 mL of the media with 1 g/L phosphate concentration. Three 100 mL aliquots were collected from each sample and were incubated for 24 h.

### 2.2. Enzymatic Assays

Acid phosphatase activity (ACP) was measured according to the previously developed method [17]. To 800 µL of 0.1 M Na-citrate buffer (pH 4.5) was added 100 µL of yeast cell suspension and 100 µL of 0.15 M paranitrophenyl phosphate. Acid phosphatase catalyzes the cleavage of the phosphate group from paranitrophenyl phosphate and its conversion to paranitrophenol. The reaction mixture was stirred and incubated for 20 min at 30 °C. Then, 500 μL of 1 M NaOH was added to stop the reaction. The specific activity of acid phosphatase was determined as the ratio of the optical density of paranitrophenol at 410 nm to the optical density of the original cell suspension at 600 nm. In the experiment, 3 biological replicates were used for each concentration (0 g/L and 1 g/L).

### 2.3. RNA-Sequencing

The YeaStar RNA Kit (cat. #R1002, Zymo Research, Tustin, CA, USA) was used for RNA isolation from yeast. Instead of a YR Digestion buffer, a buffer containing sorbitol (1 M/L), EDTA (0.1 M/L, pH 7.4), and 0.1% β-mercaptoethanol was used. Up to 2% β-mercaptoethanol was additionally added to the YR lysis buffer. Such modifications, in contrast to the methodology proposed in the kit, allowed the isolation of high-quality RNA samples from yeast cells.

Samples were treated with DNAase I (Thermo Fisher Scientific, Waltham, MO, USA) and purified using the CleanRNA Standard kit (Evrogen, Moscow, Russia). The quality of isolated RNA was assessed using agarose gel-electrophoresis.

Libraries were obtained from isolating RNA samples using QuantSeq 3′ mRNA-Seq Library Prep Kit FWD for Illumina (cat. #015.96, Lexogen, Vienna, Austria).

Libraries were qualitatively and analyzed by a Nanodrop spectrophotometer 2000c (Thermo Fisher Scientific, Waltham, MO, USA) and 1% agarose gel-electrophoresis. The pool of libraries was sequenced on an Illumina MiSeq (single read, 300 bp) at Evrogen (Moscow, Russia).

### 2.4. Bioinformatic Analysis

Filtering of the obtained reads by quality and removal of adapter sequences was performed using the Trimmomatic program (version 0.36) [18]. The quality of filtered reads was checked using the FastQC program (version 0.11.9) [19]. The reads were aligned to the yeast *K. phaffii* reference genome (ASM2700v1) and annotated from the NCBI (National Centre for Biotechnology Information) database. Alignments were performed by the hisat-2 (version 2.2.1) program with standard parameters [20]. Then, aligned reads were counted using the featureCounts program (version 2.0.1) [21]. R language version 3.6.3 and DESeq2 library version 1.24.0 were used to analyze differential gene expression [22]. Genes with a *p*-value less than 0.05 and log2FoldChange greater than 0.5 by modulus were taken into further analysis. The BLAST (version 2.10.0) algorithm was used to align amino acid sequences [23]. The number of resulting reads for each sample identified by RNA-sequencing is presented in Supplementary Materials Table S2.

### 2.5. Gene Nomenclature

For annotated *K. phaffii* genes, names were acquired from published studies. Other *K. phaffii* genes that are orthologs of the *S. cerevisiae* genes were found using BLAST (version 2.10.0) analysis. “*Kp*” index was added to the name of these genes in order to distinguish them from *S. cerevisiae* ones. Protein names have the letter “*p*” added to them.

## 3. Results

### 3.1. Influence of the Phosphate Starvation on Gene Expression in Yeast K. phaffii

**Figure 1:**
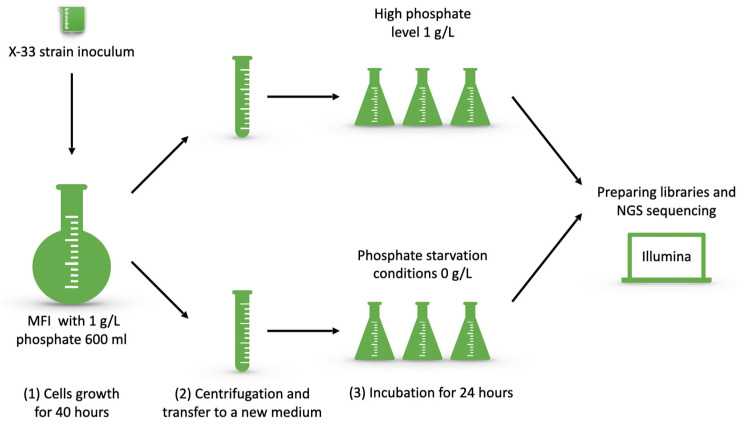
Scheme of cultivation process.

In this research, the prototrophic yeast strain X-33 was taken for transcriptome analysis. The process was carried out in two steps. First, the inoculum of strain X-33 was transferred to MFI medium. It had glycerol as a carbon source and 1 g/L of phosphate for biomass growth. The cells were incubated for 40 h. Then, the cells were collected by centrifugation and were transferred to MFI media, with either high phosphate (1 g/L) or no phosphate source (0 g/L). Three replicates were performed. After 24 h, total RNA was extracted from the cells for further library preparation (Figure 1).

The activity of acid phosphatases was measured to test the response of cells to phosphate starvation. The specific enzyme activity was calculated for each sample using quantitative technologies [17]. According to the data obtained, cells responded to the absence of phosphate in the medium under experimental conditions. They actively synthesized acid phosphatases (Table S3 in Supplementary Materials). The mean enzyme activity in cells, under phosphate starvation, was 1.53 ACP units. The control cultures at high phosphate showed no such effect (0.16 ACP specific activity units).

The transcriptome analysis revealed significant changes in the expression of 491 genes in phosphate deficiency conditions. This is about 9% of the total number of protein-coding genes in yeast *K. phaffii.* Increased expression levels were observed for 197 genes, and 294 genes were downregulated. We aligned the amino acid sequences of the identified genes with those from the yeast *S. cerevisiae* proteome. A list of these differentially expressed genes is presented in Tables S2 and S4 in the Supplementary Materials.

### 3.2. The Phosphate Starvation Influences Genes Involved in Polyphosphate Metabolism

The expression of phosphate transporter and phosphatase genes was increased under phosphate starvation. The high-affinity phosphate transporter gene *PHO89* (*PAS_chr2-1_0235*) was activated. We found that yeast *K. phaffii* putatively has two genes for the Pho84p transporter. Interestingly, one gene, *KpPHO84-1* (*PAS_chr4_0337*), is upregulated during phosphate starvation. The other gene, *KpPHO84-2* (*PAS_chr3_0141*)*,* was found to be strongly repressed under the experimental conditions.

A significant number of overexpressed genes include genes whose proteins have phosphatase activity. Under phosphate starvation, we observed activation of the acid phosphatase gene *PHO1* (*PAS_chr2-1_0103*). Its protein is secreted into the extracellular space and hydrolyses phosphorus-containing compounds. These data are corresponded by measurement of acid phosphatase enzyme activity. The effects of the response of *K. phaffii* cells to a phosphate deficiency are confirmed at both the mRNA and protein levels. Among other genes encoding proteins with phosphatase activity, the phosphorylcholine phosphodiesterase gene was upregulated (*PAS_chr2-2_0314*). This enzyme hydrolyses glycerophosphocholine into glycerol-3-phosphate and choline.

**Figure 2:**
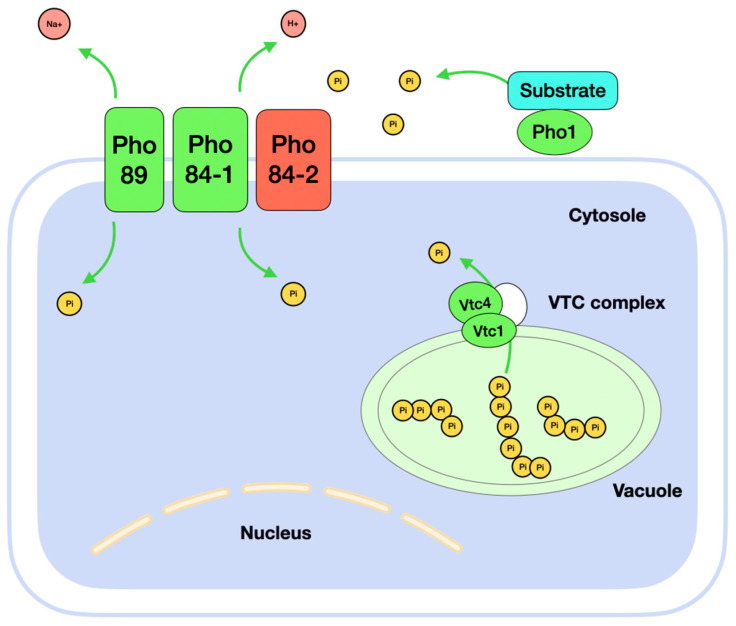
Scheme of phosphate transport and phosphate release in yeast *K. phaffii*. Pi is phosphate ion, which is free in solution. The proteins where genes are activated under phosphate starvation conditions are indicated in green. The proteins where genes are repressed under phosphate starvation conditions are indicated in red.

In conditions of phosphate deficiency, yeast cells restore the macronutrient by mobilizing intracellular phosphorus reserves. These are represented by polyphosphate molecules. Our data for *K. phaffii* showed that, under phosphate starvation, three of the five most inducible genes were the orthologs of VTC (vacuolar chaperone complex) components in the yeast *S. cerevisiae.* The Vtc1p is responsible for transporting polyphosphate across the vacuolar membrane, while the Vtc2p, Vtc3p, and Vtc4p have a regulatory function. The complex is localized in the vacuolar membrane. Its main function is to maintain the structure of the V-ATPase and the fusion of the vacuolar membrane with the membrane organelles of the cell [24]. We observed the activation of the *KpVTC4* (*PAS_chr2-2_0420*) and *KpVTC1* (*PAS_chr4_0290*) genes in our study. They encode components of the Vtc4p and Vtc1p chaperone complexes, respectively (Figure 2).

### 3.3. The Phosphate Starvation Influences Genes Involved in Carbon Metabolism and General Cellular Processes

Phosphate metabolism is part of cellular metabolism. It is closely related to central carbon metabolism [25]. Genes involved in key processes of glycolysis and gluconeogenesis showed a significant increase in expression. In order to maintain the phosphate concentration in the cells, the processes were mainly redirected to reactions that release inorganic phosphate. We found an increase in the mRNA levels of three genes: phosphoglucoisomerase (*PAS_chr3_0456*), glyceraldehyde-3-phosphate dehydrogenase (*PAS_chr2-1_0437*), and fructose-1,6-bisphosphate aldolase (*PAS_chr1-1_0072*).

In addition, phosphate deficiency affected the Krebs cycle, glycolysis, and gluconeogenesis. The gene transcription of the aconitase *KpACO1* (*PAS_chr1-3_0104*) and the isocitrate lyase *KpICL1* (*PAS_chr1-4_0338*) was activated. The pyruvate carboxylase gene isoform *KpPYC2* (*PAS_chr2-2_0024*) showed increased expression. This enzyme catalyzes the conversion of pyruvate to oxaloacetate. In addition, the transcription of genes related to mitochondrial metabolic processes was repressed. We found a general repression of transcription of genes involved in oxidative phosphorylation. In particular, there were genes for subunits of the electron transfer chain (II, III, IV complexes) and ATP synthase components (*PAS_chr2-2_0165, PAS_chr4_0520, PAS_chr2-2_0265*). Among the activated genes, there were many genes related to the processes of defense against oxidative stress in cells. We found activation of glutathione transferase genes (*PAS_chr2-1_0315*), superoxide dismutase genes (*PAS_chr1-4_0071*), and catalase genes (*PAS_chr2-2_0131*).

We observed a significant change in the mRNA levels of genes that control starch and glycogen metabolism. These polymers in yeast cells provide energy storage and mobilization under stress conditions. Increased expression was found for glycogen phosphorylase (*PAS_chr2-1_0173*) and UDP-glucose phosphorylase (*PAS_chr1-3_0122*) genes. These enzymes catalyze the reaction of glycogen breakdown to glucose. Activation was also detected for two genes: glycogen synthase and starch synthase genes (*PAS_chr3_0286, PAS_chr3_0781*). The proteins of the *LRA3* and *LRA4* genes are key to the rhamnose utilization pathway. They also showed high expression levels in the absence of phosphate in the medium [26].

### 3.4. The Phosphate Starvation Influences Genes Involved in Fatty Acids Metabolism

Phosphate deficiency had a significant effect on fatty acid metabolism. Fatty acid metabolism is divided into catabolic and anabolic processes. The data showed that phosphate starvation did not have a strong effect on the biosynthetic processes in *K. phaffii* cells. However, a significant repression was observed for the genes of beta-oxidation enzymes.

**Figure 3:**
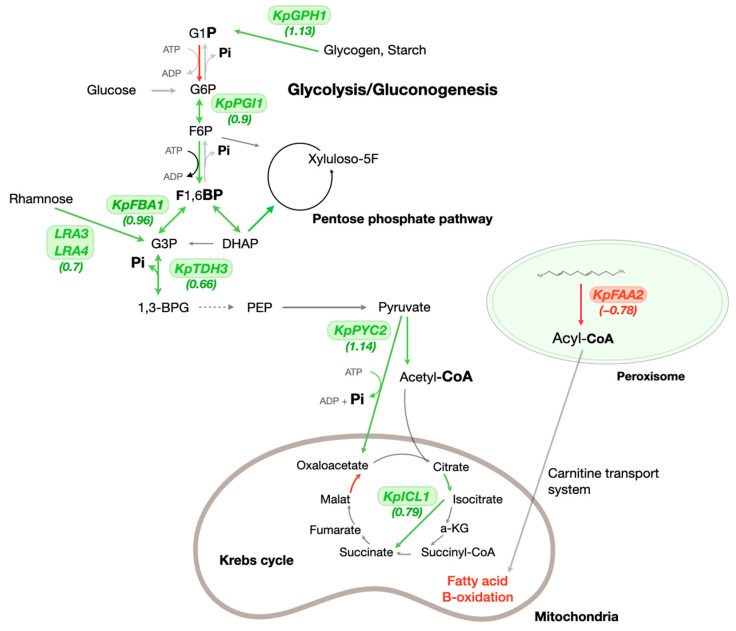
Schematic of specific metabolic pathways in the yeast *K. phaffii* based on information from the KEGG database. Upregulated genes are marked in green and are located above the corresponding reactions. Downregulated genes are highlighted in red and are located above the corresponding reactions. Reactions involving enzymes whose genes do not show increased expression under macronutrient deficiency are highlighted in gray. LFG values (change in expression between two samples) are shown in parentheses.

Repression was shown for the *KpFAA2* gene (*PAS_chr4_0352*). The acyl-CoA synthetase enzyme encoded by *KpFAA2* catalyzes the reaction of acyl-CoA molecule formation. This reaction activates fatty acids. The genes for the ketoacyl-CoA thiolase *KpFOX2* (*PAS_chr2-2_0267*) and the acetyl-CoA acyltransferase *KpERG10* (*PAS_chr3_0176*) were downregulated. The genes encode enzymes that act in the mitochondrial matrix and catalyze reactions to form acetyl-CoA and a truncated acyl-CoA. The acyl-CoA is recycled to the oxidative process (Figure 3).

### 3.5. The Phosphate Starvation Influences Genes Involved in Amino Acid Metabolism and Translation Processes

Phosphate starvation affects amino acid metabolism. In *K. phaffii* yeast cells, phosphate starvation increased the expression of genes involved in arginine metabolism. The amino acid degradation was shown to involve activation of the arginase gene *KpCAR1* (*PAS_chr4_0684*). Arginase is the last enzyme in the urea cycle, and it catalyzes the formation of ornithine by hydrolyzing the arginine molecule. In phosphate deficiency, the threonine deaminase gene *KpILV1* (*PAS_chr1-4_0243*) was activated. KpIlv1p ensures the deamination of threonine to 2-oxobutanoate molecules. On the contrary, genes encoding proteins involved in transamination reactions showed decreased expression levels. We observed the repression of alanine transaminase (*PAS_chr3_0482*) and aspartate aminotransferase (*PAS_chr1-1_0200*) genes.

In response to phosphate deficiency in *K. phaffii*, the mRNA levels of cysteine and methionine biosynthesis genes changed. A decrease in the expression level was found for genes responsible for synthesis and activation of the sulfate group (ATP sulfurylase gene *PAS_chr1-4_0253* and adenylylsulfate kinase gene *PAS_chr1-4_0253*). We found repression of genes involved in amino acid biosynthesis: *KpCYS3* (*PAS_chr4_0330*) and *KpMET6* (*PAS_chr3_1084*).

**Figure 4:**
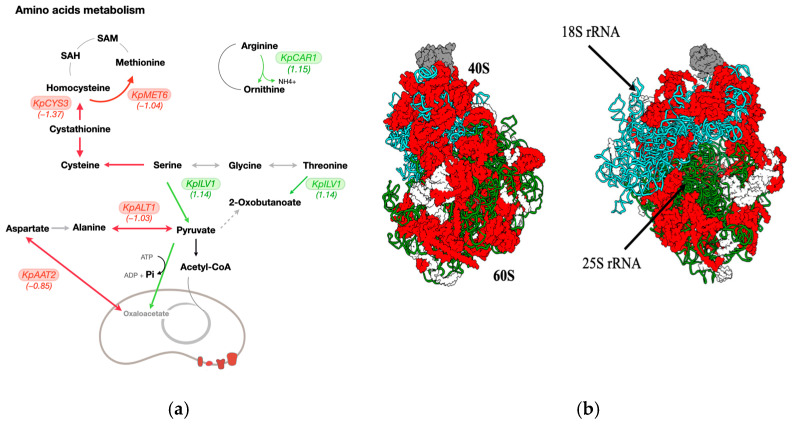
(**a**) Scheme of amino acid metabolism in the yeast *K. phaffii* based on information from the KEGG database. Upregulated genes are marked in green and are located above the corresponding reactions. Downregulated genes are highlighted in green and are located above the corresponding reactions. Reactions involving enzymes whose genes do not show increased expression under nutrient starvation are highlighted in gray. (**b**) Scheme of the yeast ribosome structure. Ribosomal proteins of downregulated genes are highlighted in red. LFG values (change in expression between two samples) are shown in parentheses.

The largest group of downregulated genes under phosphate starvation conditions was genes of protein biosynthetic processes. More than 75 downregulated genes encode different components of ribosome subunits. The yeast ribosome consists of two subunits [27]. The 60S subunit contains 3 RNA molecules and more than 46 protein molecules; the small 40S subunit has only 1 RNA and contains 33 protein molecules. In the yeast *K. phaffii,* the expression of the genes for the small and large ribosomal subunit proteins was reduced in the absence of phosphate (Figure 4).

### 3.6. The Phosphate Starvation Influences Genes Involved in Metabolism of Purine and Pyrimidine Nucleotides

**Figure 5:**
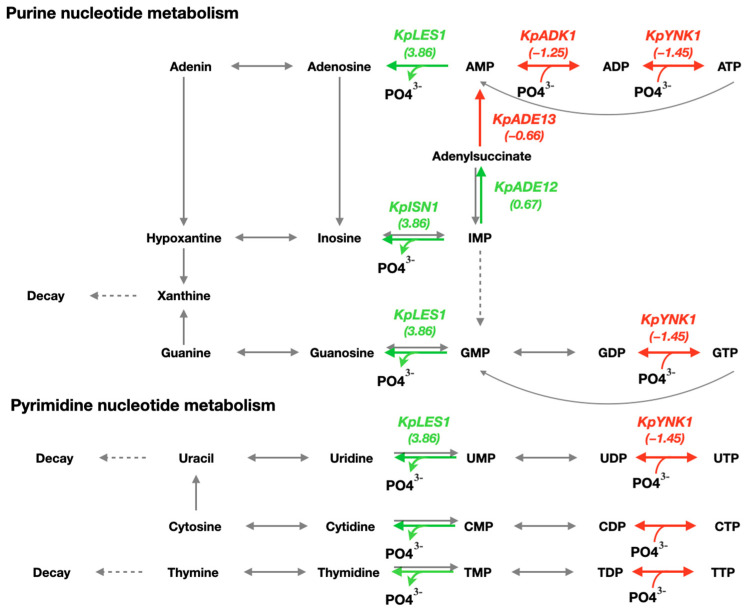
Scheme of the nucleotide metabolism of the yeast *K. phaffii* based on information from the KEGG database. Upregulated genes are marked in green and are located above the corresponding reactions. Downregulated genes are highlighted in red and are located above the corresponding reactions. Reactions involving enzymes whose genes do not show increased expression under nutrient deficiency are highlighted in gray. LFG values (change in expression between two samples) are shown in parentheses.

Nucleotide molecules contain phosphate groups. Therefore, under conditions of phosphate deficiency, their metabolism was altered by the activation of genes responsible for the degradation of nucleotide molecules. We observed an increase in the expression of 5′-nucleosidase genes (ex.: *PAS_chr2-1_0685*). A decrease in transcript levels was observed for the nucleoside diphosphate and triphosphate kinase *KpYNK1* gene (*PAS_chr2-2_0059*). The enzyme catalyzes reactions for the synthesis of nucleotide molecules, which are high-energy phosphate acceptors in cells. We found suppressed gene transcription of key purine metabolism enzymes. These are adenylate kinase *KpADK1* (*PAS_chr3_0257*) and adenylosuccinate lyase *KpADE13* (*PAS_chr2-2_0329*) genes (Figure 5). 

## 4. Discussion

The regulation and maintenance of phosphate homeostasis is carried out by a large number of proteins. They release and transport phosphate within the cell and mobilize phosphorus reserves. Phosphate metabolism is thought to be closely linked to cellular transport, carbohydrate and lipid metabolism, and cellular responses to stress and chemical agents. The complexity of phosphate concentration regulation has been investigated in *S. cerevisiae, Cryptococcus neoformans,* and *Schizosaccharomyces pombe* [28,29]. The putative number of genes involved in phosphate metabolism varies from 100 to 130 genes in these organisms. This fact underscores the extreme importance of phosphate metabolism in cell life.

Common elements of the PHO pathway have been found in many fungal species, including methylotrophic yeasts. Research has demonstrated that the key elements of the phosphate regulatory system found in *S. cerevisiae* are also present in methylotrophic yeast species *Hansenula polymorpha* [30]. This suggests that the phosphate pathway has similar features in fungi that are evolutionarily distant from *S. cerevisiae*. The PHO pathway shows similarities in many yeast species. However, some elements of phosphate metabolism regulation are quite specific and are determined by the species of the organism. The study and comparison of pathways regulating phosphate metabolism in different yeast species helps us to better understand the phosphate balance in cells.

Transcriptome analysis was performed to investigate the effect of phosphate deprivation on gene expression in *K. phaffii* cells. We found upregulated expression of 197 genes and downregulated expression of 300 genes.

Phosphate metabolism in yeast cells is a multicomponent and complex process. Enzymes such as phosphatases, polyphosphatases, permeases, phosphodiesterases, and polyphosphate kinases play an important role in phosphate balance [12]. These enzymes maintain the intracellular concentration of phosphorus in response to external conditions. At the same time, the activity of these enzymes is regulated by the extracellular concentration of inorganic phosphate (Pi). Many enzymes of phosphate metabolism are part of the specific PHO pathway. Yeast cells respond to Pi deficiency by extracting Pi from phosphorus-containing organic molecules. For this purpose, the cells activate the synthesis of specific enzymes, phosphatases, and high-affinity phosphate transporters. Enzyme phosphatases, secreted on the cell surface, hydrolyze phosphodiester bonds in organic molecules. Transporters provide efficient, intracellular transport of Pi [13].

The expression of phosphate metabolism genes was significantly altered. The genes for the high-affinity phosphate transporters Pho89p and Pho84p were among the most strongly activated genes. We identified two genes encoding the high-affinity phosphate transporters Pho84-1p and Pho84-2p in *K. phaffii* cells. Under phosphate starvation conditions, we observed a significant activation of the *KpPHO84-1* gene. The gene encoding the Pho84-2p was strongly repressed. The amino acid sequence similarity between Pho84-1p and *S. cerevisiae* Pho84p is 95%, while the similarity between *K. phaffii* Pho84-2p and *S. cerevisiae* Pho84p is 97%. An interesting observation is that two putative high-affinity phosphate transporter genes were both strongly repressed under alkalizing conditions [31]. It can be assumed that the phosphate transporter genes *PHO84-1* and *PHO84-2* have a similar origin but show different regulation in response to different conditions.

In the yeast *S. cerevisiae*, the Pho84p transporter has been shown to play a role in manganese homeostasis. It also acts as a low-affinity metal transporter [32]. In the data obtained, high- and low-affinity zinc transporter genes (*PAS_chr4_0516* and *PAS_chr3_0516*) are among the most repressed genes in phosphate deficiency. Manganese and zinc ions are essential for the function of many enzymes in cells. However, an excess of these metal ions can lead to cell damage, such as DNA damage during the replication process. Therefore, it is very important to strictly regulate the concentration of metal ions in the cell and maintain their homeostasis. In our study, we assume that the functions of phosphate and manganese transporters in *K. phaffii* yeast cells can be performed by different proteins, Pho84-1p and Pho84-2p.

Phosphate metabolism is integrated into the overall metabolism of cells. Accordingly, the systems responsible for regulating phosphate metabolism are integrated into the general regulatory network. Cells are able to restore phosphate homeostasis by regulating carbon metabolism in the metabolic network. We found a significant upregulation of genes involved in key processes of glycolysis and gluconeogenesis (*PAS_chr3_0456, PAS_chr2-1_0437, PAS_chr1-1_0072*). Many of the reactions involved are related to the conversion of compounds containing a phosphate group. They can be thought of as alternative sources of phosphorus. Depending on the direction, the reaction either consumes or releases a phosphate molecule. In this case, we assume that under phosphate starvation in *K. phaffii* cells, the reactions of carbon metabolism are preferentially redirected towards the formation of glucose. This results in the release of inorganic phosphate molecules.

Studies of stress-induced gene expression in yeast show that many proteins are induced by stress [33,34]. In our work, transcriptome analysis revealed that phosphate deprivation was closely related to the stress response of *K. phaffii* cells. It induced the activation of many genes that are involved in the defense against oxidative and replicative stress. A significant expression level decrease in genes involved in ribosome biogenesis and transport RNA synthesis was observed. This corresponded to a general suppression of ribosomal protein and translation initiation and elongation factor gene expression (*PAS_chr1-4_049, PAS_chr3_0701*). A large cluster of downregulated genes consisted of mitochondrial genes, the most repressed of which were mitochondrial electron transfer chain components (*PAS_chr2-2_0265, PAS_chr3_0997*). The data obtained are consistent with previous studies on the response of *S. cerevisiae* and *S. pombe* yeast cells to various stresses [35].

The yeast response to phosphate starvation within the same biochemical process proved quite heterogeneous. For example, phosphate starvation had a significant effect on the amino acid metabolism processes. We observed a repression of the alanine transaminase (*PAS_chr3_0482*) and aspartate aminotransferase (*PAS_chr1-1_0200*) genes. These enzymes are involved in the anabolism and catabolism of alanine and aspartate, respectively. The yeast *S. cerevisiae* has orthologs of the transaminases Alt1p and Aat2p [36]. The reduced transcript levels of the genes can be explained by the fact that the coenzyme pyridoxal phosphate is required for the transamination reactions to proceed. Due to the presence of a phosphate group in the coenzyme molecule, under conditions of phosphate limitation, the processes of pyridoxal phosphate synthesis may decrease or the processes of its cleavage in the cell may increase. On the other hand, the activation of the arginase gene *KpCAR1*, which is involved in the degradation of the amino acid, was observed. Arginase is the final enzyme in the urea cycle. It catalyzes the formation of ornithine by hydrolyzing the arginine molecule. There was also activation of the threonine deaminase gene (*KpILV1*), which catalyzes the deamination of threonine to a 2-oxobutanoate molecule. In contrast to transamination, deamination reactions proceed by releasing ammonia and decreasing the overall number of amino acid molecules.

In response to phosphate starvation, genes associated with the catabolism of nitrogenous bases are activated. We hypothesize that this may affect changes in the concentration of ATP molecules. ATP molecules are essential for many reactions. Thus, we observed the suppression of genes whose proteins are involved in reactions that consume ATP molecules. For example, the data obtained showed a decrease in the expression of cysteine and methionine biosynthesis genes in response to phosphate deficiency. Repressed genes contain the genes responsible for synthesizing and activating the sulfate group (ATP sulfurylase gene *PAS_chr1-4_0253*, adenylylsulfate kinase gene *PAS_chr1-4_0253*). An ATP molecule is also required for the synthesis of SAM, a coenzyme necessary for the transfer of methyl groups.

An interesting observation was that, under starvation, the transcription of genes encoding key purine metabolism enzymes—adenylate kinase (*KpADK1*) and adenylosuccinate lyase (*KpADE13*)—was reduced. Adenylate kinase in yeast is responsible for synthesizing ATP from the molecule adenosine monophosphate. In addition to the formation of adenosine monophosphate from adenylosuccinate, the enzyme adenylosuccinate lyase catalyzes the conversion of the carboxamide SAICA to the ribonucleotide AICAR [37]. Previous studies have shown that both enzymes are involved in the coregulation of phosphate and purine metabolism in the yeast *S. cerevisiae*. Adenylate kinase has been shown to be a potent stimulator of *PHO5* gene expression and likely influences regulation of the PHO pathway through components of signaling cascades [38]. Our data suggest a similar complex relationship between nucleotide biosynthesis and the phosphate metabolic system in the yeast *K. phaffii*.

The observed suppression of fatty acid metabolism enzyme gene expression under phosphate limitation can be explained by a decrease in the concentration of CoA molecules with up to three phosphate groups. As the primary energy source in the tricarboxylic acid cycle, CoA plays a central role in the overall metabolism of cells. Both suppression of its formation in glycolysis and increase in its cleavage processes can be associated with a decrease in coenzyme A content. Also, the decreased expression levels of genes involved in mitochondrial fatty acid oxidation processes are consistent with the overall repression of mitochondrial genes.

Changes in mRNA levels do not always reflect all aspects of the cellular response to environmental factors. Regulation at the transcript level is not the only way to control gene expression. While transcriptome analysis cannot provide a complete picture of cellular metabolism, it does provide important information for understanding the mechanisms of the cellular response to phosphate starvation. Transcriptome analysis is a crucial part of our research, providing a solid foundation for further experiments.

We expect the data to be both fundamental and practical. For example, we can use phosphate metabolism promoters for recombinant synthesis as an alternative to methanol metabolism gene promoters [39]. Therefore, understanding the mechanisms of response to phosphate starvation is an important issue.

Various strains of the yeast *K. phaffii* are used in biotechnology [40]. Despite the assumption of common origin, they can differ in a number of characteristics, including growth rate, proteolytic activity, and transformation efficiency. These characteristics are primarily related to features of their cell wall and amino acid metabolism or methanol utilization mechanisms [41]. A thorough comparison of these strains reveals their unique features and potential for biotechnological processes. This article analyzed the transcriptome response of yeast *K. phaffii* cells using the X-33 strain as an example. The X-33 strain is one of the most common in biotechnology, used extensively for producing recombinant proteins. Future studies will examine other strains of the yeast *K. phaffii*.

## 5. Conclusions

Phosphate metabolism and its regulation are well studied in yeast *S. cerevisiae*, one of the main eukaryotic model organisms. However, *S. cerevisiae* yeast has a number of unique features related to the organization of its genome and peculiarities of some biochemical processes [42]. It is essential to study biochemical pathways and ways of their regulation in other species. Comparing biological processes across different yeast groups is crucial for understanding evolutionary processes and studying metabolic system regulation in eukaryotic cells. The yeast *K. phaffii* differs from *S. cerevisiae* in that it is methylotrophic and strictly aerobic [7]. In this study, we analyzed changes in gene mRNA levels in yeast *K. phaffii* under phosphate starvation.

Our findings clearly indicate that the absence of this macronutrient in the medium led to the activation of classical mechanisms of phosphate homeostasis regulation. Specifically, we observed the activation of PHO pathway components, including phosphatases, polyphosphatase, inorganic phosphate transporters, and enzymes with proteolytic and hydrolase activity. Cells compensated for the lack of the macronutrient by degrading various phosphate-containing organic molecules, such as phospholipids and components of subcellular organelles. Absence of phosphate is a stressor with a strong effect on yeast cells. Phosphate starvation represses a large number of genes involved in ribosome biogenesis and protein synthesis, as well as mitochondrial ATP molecule synthesis.

In contrast, phosphate metabolism and its regulation are closely related to the cells’ general metabolism. The response to phosphate starvation affects metabolic processes in yeast cells in general, and it is heterogeneous. We observed redirection of carbon metabolism processes, suppression of fatty acid beta-oxidation processes, and activation of nucleotide degradation. In phosphate starvation, processes requiring phosphorus molecules were mostly inhibited. The transcription of genes for enzymes involved in releasing inorganic phosphate molecules was activated.

Our study definitively shows a significant effect of phosphate deficiency on the general metabolism in yeast cells. The analysis focused on the known data of yeast *S. cerevisiae* and compared the data obtained with these microorganisms. The key components were similar, but we also observed differences, particularly regarding the activation of phosphate transporter gene expression.

*K. phaffii* is a highly utilized organism in both the production of recombinant products and synthetic biology, where it plays a crucial role in the synthesis of diverse chemical compounds and biofuels [43]. Fatty acids, sugar alcohols, and terpenoids are actively synthesized using *K. phaffii*. However, the methods of synthesis are not always suitable for large-scale production [44]. A significant number of technologies are being developed to optimize the synthesis of chemical compounds in these organisms.

We have discovered several effects that influence these synthetic processes. Specifically, we have found that suppressing the beta-oxidation of fatty acids can lead to the synthesis of byproducts, increased levels of intermediate derivatives, or the accumulation of metabolites for the synthesis of desired compounds. The data obtained are crucial for understanding the specificity of biochemical processes in *K. phaffii* cells and may be useful for modifying and optimizing product biosynthetic pathways. This gives us control over biochemical processes and the ability to efficiently redirect reactions for the production of desired compounds.

## Data Availability

The original contributions presented in the study are included in the article/Supplementary Material, further inquiries can be directed to the corresponding authors.

## Supplementary Materials

The following supporting information can be downloaded at: https://www.mdpi.com/article/10.3390/microorganisms13010039/s1, Table S1: The composition of 2500× solutions of vitamins and trace elements per 100 mL; Table S2: The results of the differential gene expression analysis using DESeq2; Table S3: Results of acid phosphatase activity and optical density measurements for calculating specific activity; Table S4: The results of amino acid sequence alignment for repressed/activated genes of *P. pastoris* (*K. phaffii*) to *S. cerevisiae* amino acid sequence databases.
